# Assessment of the implementation context in preparation for a clinical study of machine-learning algorithms to automate the classification of digital cervical images for cervical cancer screening in resource-constrained settings

**DOI:** 10.3389/frhs.2022.1000150

**Published:** 2022-09-12

**Authors:** Delivette Castor, Rakiya Saidu, Rosalind Boa, Nomonde Mbatani, Tinashe E. M. Mutsvangwa, Jennifer Moodley, Lynette Denny, Louise Kuhn

**Affiliations:** ^1^Division of Infectious Diseases, Vagelos College of Physicians and Surgeons, Columbia University Irving Medical Center, New York, NY, United States; ^2^Department of Epidemiology, Mailman School of Public Health, Columbia University Irving Medical Center, New York, NY, United States; ^3^Department of Obstetrics and Gynaecology, University of Cape Town, Cape Town, South Africa; ^4^Groote Schuur Hospital and South African Medical Research Council, Gynaecology Cancer Research Centre, University of Cape Town, Cape Town, South Africa; ^5^Division of Biomedical Engineering, Department of Human Biology, University of Cape Town, Cape Town, South Africa; ^6^Women's Health Research Unit, School of Public Health and Family Medicine, University of Cape Town, Cape Town, South Africa; ^7^Gertrude H. Sergievsky Center, Vagelos College of Physicians and Surgeons, Columbia University Irving Medical Center, New York, NY, United States

**Keywords:** digital cervical-cancer screening implementation assessment cervical cancer, implementation science, CFIR, Automated Visual Evaluation, cervical cancer, CFIR framework, Automated Visual Evaluation of the cervix

## Abstract

**Introduction:**

We assessed the implementation context and image quality in preparation for a clinical study evaluating the effectiveness of automated visual assessment devices within cervical cancer screening of women living without and with HIV.

**Methods:**

We developed a semi-structured questionnaire based on three Consolidated Framework for Implementation Research (CFIR) domains; intervention characteristics, inner setting, and process, in Cape Town, South Africa. Between December 1, 2020, and August 6, 2021, we evaluated two devices: MobileODT handheld colposcope; and a commercially-available cell phone (Samsung A21ST). Colposcopists visually inspected cervical images for technical adequacy. Descriptive analyses were tabulated for quantitative variables, and narrative responses were summarized in the text.

**Results:**

Two colposcopists described the devices as easy to operate, without data loss. The clinical workspace and gynecological workflow were modified to incorporate devices and manage images. Providers believed either device would likely perform better than cytology under most circumstances unless the squamocolumnar junction (SCJ) were not visible, in which case cytology was expected to be better. Image quality (*N* = 75) from the MobileODT device and cell phone was comparable in terms of achieving good focus (81% vs. 84%), obtaining visibility of the squamous columnar junction (88% vs. 97%), avoiding occlusion (79% vs. 87%), and detection of lesion and range of lesion includes the upper limit (63% vs. 53%) but differed in taking photographs free of glare (100% vs. 24%).

**Conclusion:**

Novel application of the CFIR early in the conduct of the clinical study, including assessment of image quality, highlight real-world factors about intervention characteristics, inner clinical setting, and workflow process that may affect both the clinical study findings and ultimate pace of translating to clinical practice. The application and augmentation of the CFIR in this study context highlighted adaptations needed for the framework to better measure factors relevant to implementing digital interventions.

## Introduction

Global disparities in cervical cancer incidence and mortality persist. Women living in sub-Saharan Africa bear a disproportionate burden due to the failure of current cervical cancer screening programs (1). Screen-and-treat (SAT) is a strategy for cervical cancer screening that has been shown to improve outcomes and is currently recommended by the World Health Organization for low- and middle-income country (LMIC) settings (2). Recently developed smartphone technologies that utilize machine-learning algorithms to automate the classification of digital cervical images are an encouraging method with the SAT approach to address the achievement gap in LMICs (3–7). Preliminary studies suggest that Automated Visual Evaluation (AVE) devices could detect cervical cancer precursor lesions in some populations (3, 5, 7). Private companies have developed proprietary devices to obtain images and run automated classifiers to improve screening (8, 9). Other groups are using non-proprietary cell phones to take cervical photographs to integrate automated classifiers through apps or other interfaces eventually. Requirements for successful use of AVE devices highlight attributes of the device and the algorithm that affect cervical image quality, but not the clinical setting (3). Pragmatic trialists advocate for measuring these operational attributes that influence study outcomes and ultimately impact the translation of scientific innovation to practice settings, but this is rarely done (10–12). Implementation science frameworks typically used for “real-world” delivery can be applied to assess and inform implementation issues during the clinical study stage and strengthen inference about internal and external validity (13). In preparation for a clinical study to evaluate the performance characteristics of AVE methods utilizing images collected from both a proprietary mobile device and a commercially-available smartphone for cervical cancer screening among women living with HIV and women not infected with HIV, we assessed the implementation context and image quality using an implementation framework.

## Materials and methods

Our team, which included experienced colposcopists, conducted a mixed-methods study utilizing the Consolidated Framework for Implementation Research (CFIR) to examine two devices: MobileODT hand-held colposcope and the Samsung A21ST, a commercially-available cell phone. The MobileODT device is an FDA-approved hand-held colposcope that captures and stores digital photographs of the cervix and can interface with a cloud-based machine-learning algorithm to generate an automated diagnosis based on the cervical image (8, 9).

### Study setting

The study was implemented at a free-standing clinical research site, constructed out of repurposed shipping containers, on the compound of Khayelitsha Site B Primary Health Care Clinic (KPHC), a large public clinic serving a disadvantaged community on the outskirts of Cape Town, South Africa.

### Consolidated framework for implementation research

**Figure 1:**
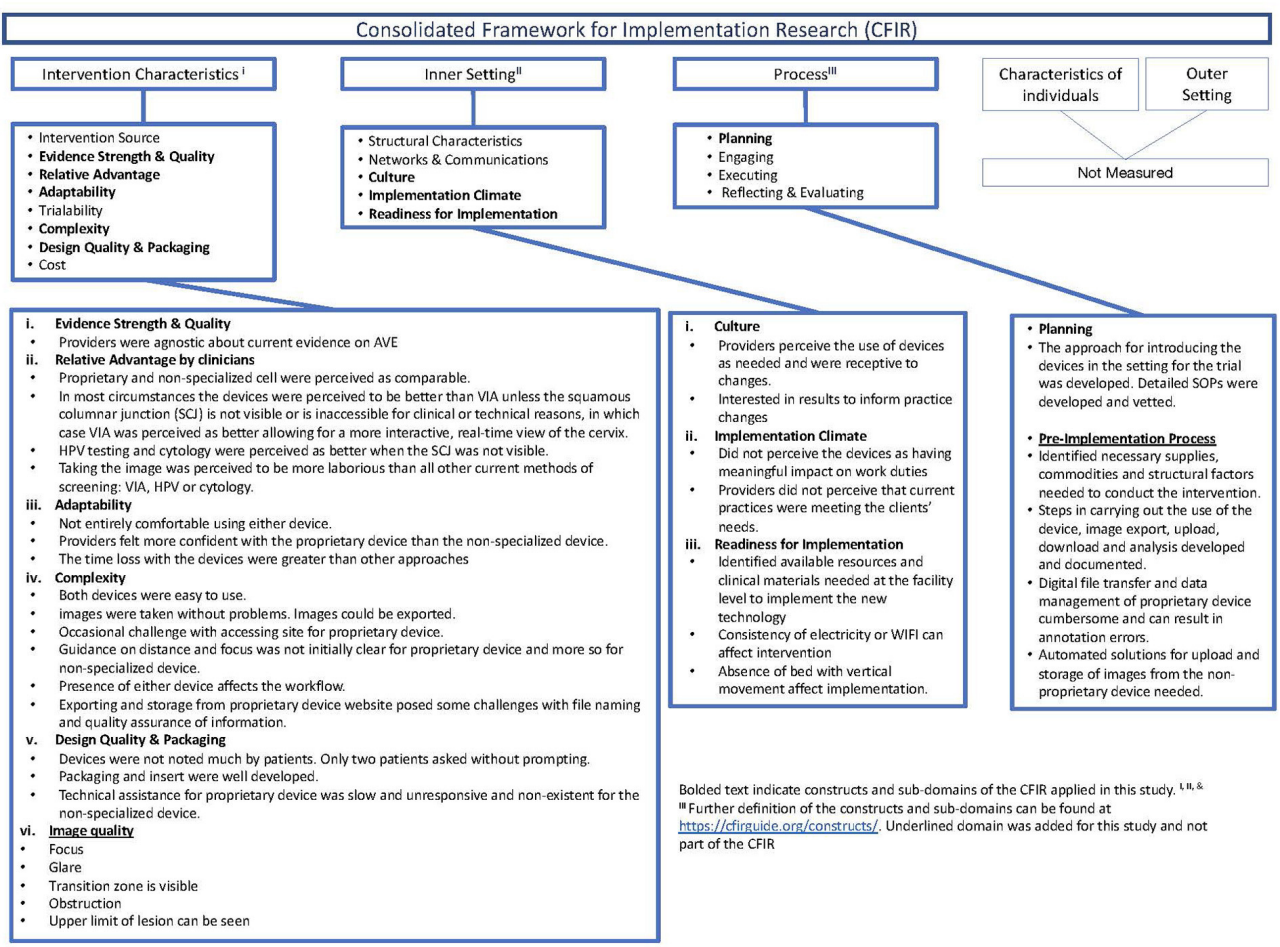
Description of the consolidated framework for implementation research (CFIR), constructs applied, and summary of findings in the preparatory phase of the clinical study.

The CFIR is an implementation science framework for assessing barriers and facilitators to the successful implementation of novel interventions through five constructs: (1) Intervention characteristics, (2) Inner setting, (3) Process, (4) Outer Setting, and (5) Characteristics of the individuals (14). Typically, the CFIR is used during the implementation phase after established effectiveness. We applied the CFIR during the preparatory phase of a clinical study and utilized selected sub-domains of three of the constructs (Figure 1). Further, we modified the CFIR to include pre-trial readiness activities, including the intervention's commodities and data management aspects. We applied four of the eight sub-domains and added a sub-domain on digital image quality within the intervention characteristics construct; adaptability, complexity, perceived strength and quality of evidence base, and relative advantage of the mobile devices compared to the other screening modalities, including visual inspection with acetic acid (VIA), cytology, and human papillomavirus (HPV) testing. We utilized the sub-domains of readiness for implementation, climate, and culture within the inner setting to assess adaptations needed to incorporate the device into the clinical space and workflow. Last, we used the planning sub-domain and added a pre-implementation sub-domain to the process construct to identify the commodities, including data transfer and management capabilities needed to acquire, manage and analyze the digital image data required to effectively screen for cervical cancer.

We developed an eighty-five-item semi-structured questionnaire. A study team member administered the semi-structured interview to two clinicians preparing to use the MobileODT and Samsung A21ST devices at the clinical research site. Responses were coded as either yes/no or using Likert scales. Providers elaborated with qualitative responses on any quantitative measures.

In addition, colposcopists visually inspected the quality of all the cervical images taken in the context of site preparation and assessed them for technical adequacy. Image quality was scored for five key attributes; (1) focus, (2) glare, (3) visibility of the squamous columnar junction (SCJ), (4) occlusion, and (5) detection of lesion and range of lesion, including the upper limit. Composite image quality scores ranged from a minimum of 0 to a maximum score of 5. Frequency counts and percentages were tabulated for quantitative categorical variables, and narrative responses were summarized in the text.

## Results

Between December 1, 2020, and August 6, 2021, two providers screened 75 women at the research site in Khayelitsha, South Africa. Results from this evaluation using the CFIR are shown in Figure 1.

### Intervention characteristics

Providers' perceptions of the relative advantage of the devices compared to standard practice interventions (i.e., VIA, HPV testing, cytology) were similar before and after use. Providers thought that either machine would outperform VIA or cytology in general. However, if lesions are present on the endocervix or the squamous columnar junction (SCJ) is not visible or inaccessible for clinical or technical reasons. Cytology was perceived as better, and VIA was perceived as comparable or better, allowing for a more interactive real-time view of the cervix. Providers agreed that HPV testing was expected to be better when the SCJ was not visible but had a divergence of opinion when comparing the perceived relative advantage of the mobile devices to HPV testing under other conditions, such as when anogenital lesions are present.

Both devices had a learning curve to be used effectively but were easy to activate, take, edit, and upload images without data loss. Documentation of the images, including correct labeling before image capture and upload and storage after image capture, required additional attention. To improve usability, the MobileODT device has some design modifications; a stand, built-in light source, autofocus feature, and handsfree activation. MobileODT automated image upload to a storage platform but required some input to ensure appropriate patient linkage before image collection. The cell phone required experimentation with external light sources and flash features. Providers used a gimbal and the “open-camera” app as strategies for a stand-and-hands-free activation for the cell phone; however, neither option was more accessible than holding the phone in their hand. A gimbal is a tool that uses motors and intelligent sensors to stabilize the camera, but the type that was purchased did not allow retention of raw digital images. Labeling, uploading, and storing the cell phone images required providers to complete additional manual steps that were not needed with the MobileODT device.

### Image quality

**Figure 2:**
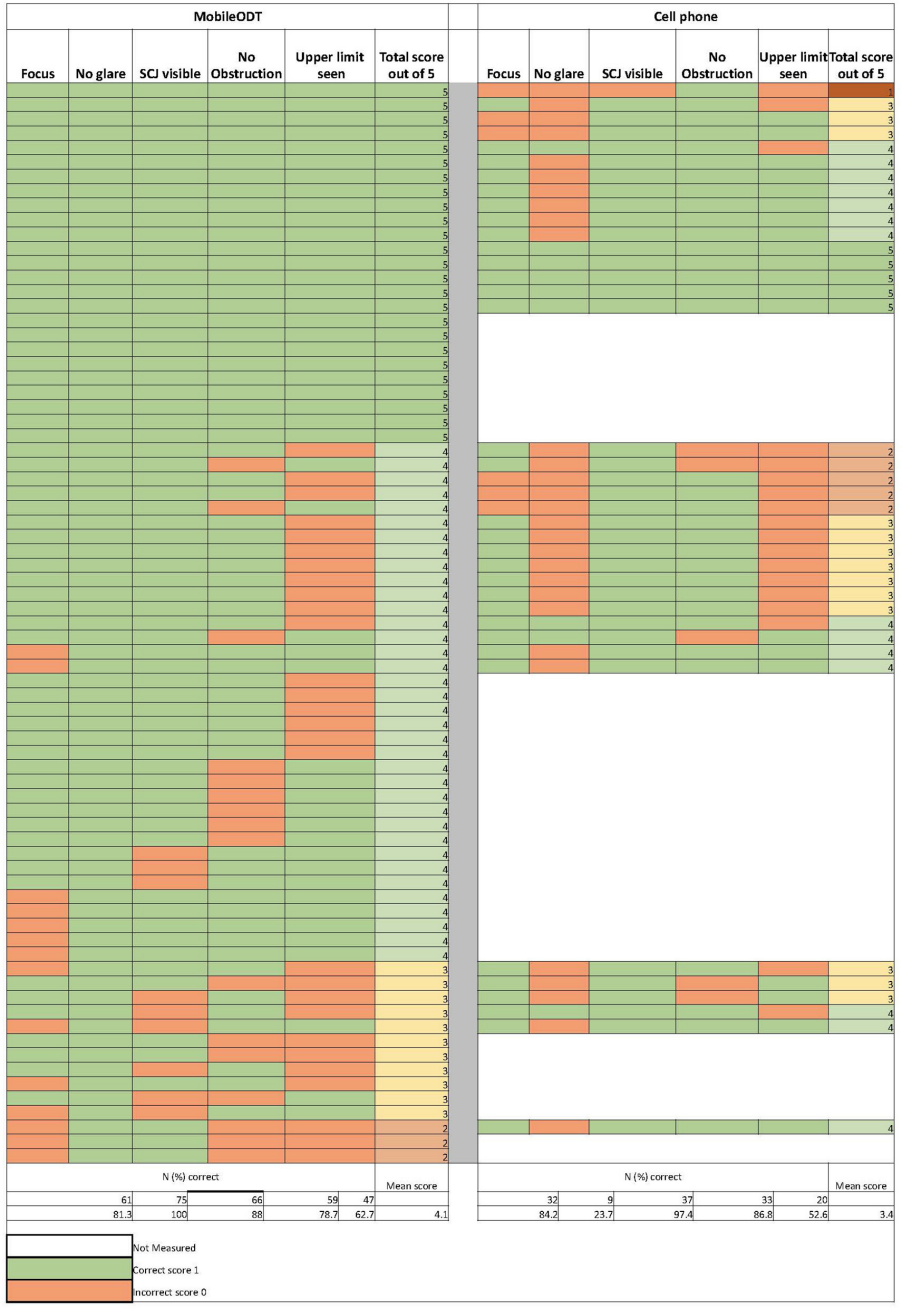
Individual results of the digital image quality scoring criteria assessments from the MobileODT device (*N* = 75) and the cell phone (*N* = 38).

Figure 2 shows individual and summarized image quality assessment scores for MobileODT (*N* = 75) and the cell phone (*N* = 38). MobileODT and the cell phone performance in terms of achieving good focus (81% vs. 84%), obtaining visibility of the SCJ (88% vs. 97%), avoiding occlusion (79% vs. 87%), and detection of the range of the lesion to include the upper limit (63% vs. 53%) was comparable. However, the MobileODT device outperformed the cell phone's function in taking photographs free of glare (100% vs. 24%). MobileODT and cell phone cumulative scores across the five image quality metrics were; 33% vs. 13% scored a five, 48% vs. 37% scored a four, 15% vs. 34% scored a three, 0.04% vs. 13% scored a two, and 0% vs. 0.03% scored a one, respectively.

### Inner setting

The space available at the clinical research site in the exam room posed minor obstacles for using either device. We rearranged the space to create an adequate area for the device and staff to operate comfortably. Infrastructure constraints, including frequent and regular loss of electricity and WIFI, adversely affected uploading the images from both devices. A gynecological bed with vertical movement could better accommodate image capture from both devices. The clinical workflow was modified for pre-collection labeling with both devices. The cell phone required manual upload and storage.

### Process

Both devices performed reasonably well—they charged quickly, the MobileODT device linked to the website and cloud, operated with minimal issues, and posed no significant logistical challenges to the providers in taking reasonable images. About 10% of the MobileODT device used were delayed and needed a restart. MobileODT automated data upload, whereas the cell phone required the provider to upload the images in a cumbersome and time-intensive way manually. We used the “open camera” app, and subsequently used a gimbal for their “always on” feature on the cell phone because cell phone images were more affected by variation in lighting. The built-in flash, flashlight feature of the cell phone, or the gimbal's always on could not adequately mirror the consistent light source available on MobileODT. Providers modified the use of the device (e.g., take the proprietary device off the stand used to stabilize) because of anatomical variation in some patients. The MobileODT device automatically uploaded images to secure storage, but there were constraints to the proprietor's cloud. We did not test the automated classification feature and return of results of the MobileODT device in this evaluation phase.

## Discussion

Our novel application of the CFIR framework during the preparatory phase of a clinical study evaluating the classification of cervical images taken with two AVE devices revealed many domains where operational issues can affect the conduct and outcome of the clinical research. Implementation science frameworks developed for systematic evaluation in real-world settings can be utilized to assess the pragmatic aspects of experimental designs that can influence study outcomes through a theory-based approach (13). We modified the CFIR construct on intervention characteristics to incorporate data transfer and management practices for digital images and machine learning interventions. Also, the process construct was modified to include pre-implementation materials and processes, including electrical, internet, and other digital infrastructure needed for implementation. The changes and use of the CFIR will strengthen the clinical study and could accelerate translation to public health practice.

There were several limitations and strengths to this study. Study limitations included few providers and sites in this preliminary evaluation. While providers gleaned some patient feedback, we did not directly assess patient perspectives in this evaluation. We evaluated two devices simultaneously. Using a less costly, commercially-available, non-specialized cell phone may overcome some economic barriers to broader reach and better coverage in health systems of LMICs.

Conversely, the proprietary device successfully addressed some challenges with taking and storing high-quality digital images. The device evaluation was conducted while clinical services were adapted to mitigate COVID-19 risk. The unique period resulted in frequent consultations within the clinical team and rapid identification and sharing of best practices, which heightened the organization's readiness for practice change. The evaluation was conducted at a research site on a public primary health care clinic compound in a deeply-impoverished, densely-populated urban community in Cape Town. The cervical cancer screening program setting is representative of other resource-limited locations where this approach is likely to be implemented if found to be effective. The assessment likely identified implementation challenges that must be confronted in other public care, resource-constrained environments.

We applied the CFIR to examine implementation factors at this early stage of the clinical study. We selected the CFIR because it is an implementation and scaling framework we intend to apply throughout the research and development process. Should the device show clinical effectiveness, we will use the CFIR in its classical form during the introduction and scale-up phase of implementing a health intervention. In this evaluation, we utilized three of the five domains of the framework. We modified two domains used to adequately measure the program, facility, and provider-level preparation needed to support the digital devices. Should the devices prove effective, the two additional domains not included in this evaluation, outer setting and individual characteristics, would also need to be evaluated.

The context domain includes an assessment of efforts to support policy changes and the broader implementation context that becomes relevant once the intervention is implemented in a real-world setting. For instance, the device should be incorporated into national guidelines. Implementation plans will need to be modified or developed. The digital device should be integrated into national and local health information systems. Human resource considerations should be extended to include skilled bioinformaticians, engineers, and developers to sustain the evolving system. Costing studies to inform implementation and funding advocacy will also be necessary (15). Funding for maintaining hardware, stable electricity, the digital infrastructure, and costs for essential accessories and acquiring, managing, storing, and analyzing the data for machine learning (16). Other important global factors to address in implementing machine learning digital devices will involve complex ethics, legality, and social issues that should be anticipated and addressed (15, 17, 18).

Embedded within the broader context are other vital factors that we believe are critical for sustainable scale-up, but we could not fully assess in this study; the evolving algorithm, device, provider, and patient that would be further examined in the context of scaling that we could not adequately assess. Critically important issues relating to the algorithm and device include data quality improvements, starting with reliable data availability with validated clinical outcomes. We may reduce the likelihood of false classification due to limited data with annotated digital image data linked with histological results and other clinical, anatomical, or biological factors that may confound the digital method, sampling a higher fraction of the population. Improvements in the classifier may increase advocacy and demand for the digital method among various cervical cancer stakeholders in the health system. Also, important aspects of the machine learning device are algorithmic transparency, safeguards to minimize the harmful effects of bias, regulatory guidance, liability, and accountability. At the provider level, capacity strengthening through training on the use of the device, Machine learning, and AI, as well as alternations to the workflow, task-shifting, and sharing to improve program efficiency while addressing program quality (19–21). Individual characteristics important for scaling include assessments of end-user values and preferences. User-specific issues that will influence scaling include the use of the device concerning patient's privacy, data security, knowledge and perception of the relative advantage of the device compared to routine methods for cervical cancer screening, and patient's time (14). Further, considerations about equity in linkage and access to gynecologic care sustained patient safety with the application of the algorithm and use of the device with reinforcement through clinical expertise; and the machine learning device's impact on the patient-physician relationship (15, 17, 21).

Experience from other machine learning interventions in Africa suggests that scaling up machine learning digital methods can be supported with data demonstrating that digital aid's effectiveness, precision, and efficiency improvements as the training data expands (22). We endeavor to support scale-up at this earliest stage. We identified common issues in using any device for image capture that should be addressed as part of site preparation; space reconfiguration, labeling, data storage, and adapting to anatomic variation in patients. We also identified device-specific issues, such as an internal light source, stability, and connectivity, that would need to be addressed. The systematic image quality assessment we undertook provided insight into quality metrics that would be important to include in quality control. We were able to gauge the clinicians' confidence, comfort, and enthusiasm about the investigated intervention. The evaluation also provided benchmarks to identify and address emerging issues at the site level that may influence study findings.

## Conclusion

The CFIR and image quality assessment provide a comprehensive approach to monitoring and evaluation, and quality improvement practices with this screening tool should effectiveness be proven. Although automated diagnosis from digital cervical images is a promising approach, several operational considerations need careful attention to ensure that this potential screening modality can operate at a high standard (4).

## Data availability statement

The raw data supporting the conclusions of this article will be made available by the authors, without undue reservation.

## Ethics statement

The study was approved by the University of Cape Town #665/2020 Human Research Ethics Committee and Columbia University #AAAT0187 Institutional Review Board. The patients/participants provided their written informed consent to participate in this study.

## Author contributions

DC, LK, RS, LD, RB, and JM conceptualized and designed the study. DC, LK, and RS managed and analyzed the data. LK and LD acquired funding for this study. DC prepared the figures and tables and the first draft of the manuscript. All authors reviewed, edited, and commented on the final draft. All authors contributed to the article and approved the submitted version.

## Funding

This study was funded in part by the National Cancer Institute (R01CA254576 and R01CA250011).

## Conflict of interest

The authors declare that the research was conducted in the absence of any commercial or financial relationships that could be construed as a potential conflict of interest.

## Publisher's note

All claims expressed in this article are solely those of the authors and do not necessarily represent those of their affiliated organizations, or those of the publisher, the editors and the reviewers. Any product that may be evaluated in this article, or claim that may be made by its manufacturer, is not guaranteed or endorsed by the publisher.
